# Fe_3_O_4_ nanoparticle-coated mushroom source biomaterial for Cr(VI) polluted liquid treatment and mechanism research

**DOI:** 10.1098/rsos.171776

**Published:** 2018-05-09

**Authors:** Can Wang, Huakang Liu, Zizhao Liu, Yufeng Gao, Bin Wu, Heng Xu

**Affiliations:** Key Laboratory of Bio-resources and Eco-environment (Ministry of Education), College of Life Science, Sichuan University, Chengdu, Sichuan 610064, People's Republic of China

**Keywords:** hexavalent chromium, Fe_3_O_4_ nanoparticle, biochemical detoxication, liquid, mushroom substrate

## Abstract

*Agrocybe cylindracea* substrate–Fe_3_O_4_ (ACS–Fe_3_O_4_), a Fe_3_O_4_ nanoparticle-coated biomaterial derived from agriculture waste from mushroom cultivation, was developed to remove hexavalent chromium (Cr(VI)) from liquid. After modification, material surface became uneven with polyporous and crinkly structure which improved Cr-accommodation ability in a sound manner. Optimized by the Taguchi method, Cr(VI) removal percentage was up to 73.88 at 240 min, 40°C, pH 3, Cr(VI) concentration 200 mg l^−1^, dosage 12 g l^−1^, rpm 200. The efficient Cr(VI) removal was due to the combined effect of adsorption and redox. In addition, verification test using tannery wastewater, with removal percentage of Cr(VI) and total Cr reaching 98.35 and 95.6, provided further evidence for the efficiency and feasibility of ACS–Fe_3_O_4_. The effect of storage time of the material on Cr(VI) removal was small, which enhanced its value in practical application. Results indicated that metal removal was mainly influenced by solution concentration, adsorbent dosage and treatment time. The experimental data obtained were successfully fitted with the Langmuir isotherm model. Thermodynamic study indicated the endothermic nature of the process. The results confirmed that ACS–Fe_3_O_4_ as novel material derived from waste, with long-term stability, could be applied for heavy metal removal from wastewater and waste cycling.

## 1. Introduction

Chromium (Cr) is a toxic heavy metal widely spread into living environment and a well-known carcinogen, which is mainly from industries such as electroplating, leather tanning, textile dyeing and steel fabrication. [1,2]. The United States Environmental Protection Agency set the maximum contaminant limits as 100 µg l^−1^ for total chromium in drinking water [3]. Exposure to Cr is detrimental to human health and has been linked to carcinomas, mutations and DNA damage [4–6]. Cr(III) and Cr(VI) coexist in aquatic environment. Cr(VI), whose toxicity is hundreds of times more than that of Cr(III), could be easily accumulated in the food chain and seriously affects human physiology [7]. Conventional remediation techniques typically involve Cr(VI) precipitation as chromium iron hydroxide or chromium hydroxide or Cr(VI) transformation to Cr(III), including phytoextraction, reverse osmosis, electrodialysis, ion exchange and physical adsorption [8]. Among these technologies, physical adsorption has been widely used because of its low cost and high efficiency [9]. Many materials have been investigated to remove pollutants from liquid, including activated carbon, lignite and bentonite [10].

Among them, biomaterial has become the hottest topic in this research [11]. The removal of heavy metals by plant tissues or by-products from agricultural, industrial or pharmaceutical industry has been proved with high efficiency and low cost [12]. Rice husks, cone biomass of *Thuja orientalis* and by-product of edible mushroom have been proved efficient in metal removal because of their large quantity of adsorption sites and functional groups [13–15]. With utility for Cr(VI) treatment, certain limitations like low density and poor mechanical strength exist, and it is necessary to develop new and efficient novel adsorbent for better application in practical use [16].

Iron-based materials have received significant attention for environmental applications [17]. Iron magnetic nanoparticles are attractive for remediation as they possess high surface areas, are inexpensive, and easily separated and recovered by simply applying an external magnetic field. Bare magnetite Fe_3_O_4_ nanoparticles have been successfully applied to remediate Cr(VI)-contaminated waters [18]. The Fe(II) in magnetite can initiate the reduction of Cr(VI) to Cr(III), which results in the toxicity reduction and formation of inner-sphere surface complexes at the surface of iron oxide due to the chelation of Cr(III) and –OH groups [19]. However, they are not effective under basic conditions and the Fe(II) in magnetite is highly susceptible to auto-oxidation [20]. Besides, although its utilization is promising for water treatment, nanomaterial is susceptible to agglomeration, and bare nanoparticles can be toxic [21]. Coating of nanomaterial on organic material is an efficient strategy to reduce its toxicity and improve its stability [22].

This study aims to investigate the potential of Fe_3_O_4_ nanoparticle-coated biomaterial derived from edible mushroom substrate to remove Cr(VI) from wastewater. Spent cultivation substrate of *Agrocybe cylindracea* was selected from several mushroom substrates (*A. cylindracea*, *Lentinula edodes* and *Collybia radicata*) for Cr(VI) removal. ACS–Fe_3_O_4_ (*A. cylindracea* substrate*–*Fe_3_O_4_) was synthesized and its ability was tested and optimized. Scanning electron microscopy (SEM), energy-dispersive X-ray analysis (EDX) and Fourier transform infrared (FTIR) were deployed to analyse the chemical elements on the surface of the biomaterial. Sorption isotherms study, adsorption kinetics study and adsorption thermodynamic study were conducted to investigate the adsorption mechanism. The Taguchi method was performed to investigate the optimum operation conditions under the influence of external interference.

## 2. Material and methods

### 2.1. Selection of biosorbent

*Agrocybe cylindracea, L. edodes, C. radicata* are common edible mushrooms in China with enormous output. The spent substrates of *A. cylindracea* (ACS), *L. edodes* (LES) and *C. radicata* (CRS) were collected from edible fungus cultivation base of Shuangliu Chengdu, China (30°31′42^″^ N, 103°52′22^″^ E). The biomaterial was oven-dried at 80°C for 24 h and powdered by a sample mill (Foss Tecator, Sweden) through 0.45 mm copper sieves. It was water-washed, dried and stored in polyethylene bottles in vacuum dryer for further use. The ingredients of them are shown in table 1.

**Table 1.** Main ingredients of mushroom cultivation substrates.

substrate	cotton shell (%)	wood chips (%)	wheat bran (%)	lime (%)
*A. cylindracea*	89	—	10	1
*L. edodes*	—	79	20	1
*C. radicata*	40	39	20	1

**Figure 1.**
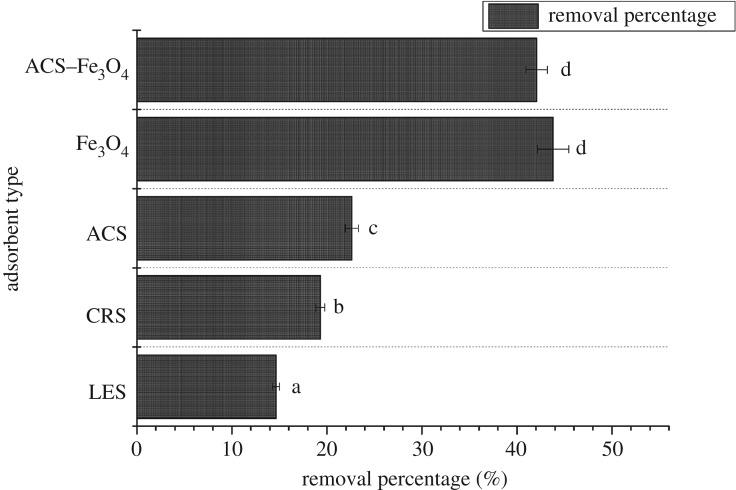
Effects of different adsorbents on Cr(VI) removal. Error bars represent the standard deviation of three samples. Columns denoted by different lowercase letters indicate significant (*p *< 0.05) difference among treatments.

Then they were tested for Cr(VI) removal from liquids. 0.20 g of biosorbent was suspended in 50 ml of 200 mg l^−1^ Cr(VI) solution in 100 ml conical flask at 25°C, 180 rpm for 24 h. As shown in figure 1, ACS had the best ability for Cr(VI) removal and it was used for the next step.

### 2.2. Preparation of ACS–Fe_3_O_4_

ACS–Fe_3_O_4_ materials were prepared according to a published method [23]. FeCl_2_·4H_2_O (3.0 g) and FeCl_3_·6H_2_O (6.1 g) were dissolved in 100 ml of water. The mixture was heated to 90°C in a 250 ml round-bottom flask equipped with a reflux condenser. The reaction solution was magnetically stirred throughout the process. To the mixture, 10 ml of 25% ammonium hydroxide and 0.50 g ACS particle (for ACS–Fe_3_O_4_ synthesis) were added rapidly and sequentially. The mixture was aged at 90 ± 5°C for an additional 30 min. The solid products were washed with water and dried to constant weight in a vacuum oven at approximately 40°C. The particles were stored in a vacuum desiccator. Pure Fe_3_O_4_ was also prepared with the same method without ACS. The ability of pure Fe_3_O_4_ and ACS–Fe_3_O_4_ for Cr(VI) removal was also tested with the same condition mentioned in §2.1.

### 2.3. Chemicals and equipment

All the chemicals and reagents used were of analytical grade (Kelong Chemical Reagent Factory, Chengdu, China). Potassium dichromate was used as adsorbate. The stock adsorbate solutions (1000 mg l^−1^ and 5000 mg l^−1^) were prepared by dissolving 2.828 and 14.140 g of potassium dichromate in 1 l ultrapure water, respectively. All working solutions were obtained by dilution. Cr(VI) concentration was determined by a spectrophotometer according to Chinese National Standard GB/T 7467-87. The total Cr was determined by a flame atomic absorption spectrometer (AA700, Perkin-Elmer, USA). The Cr(III) content was the difference between total Cr and Cr(VI) in the solution.

### 2.4. Characterization of ACS–Fe_3_O_4_

SEM (JSM-5900LV, Japan) was used to identify the surface morphology features of raw ACS and ACS–Fe_3_O_4_. The chemical elements on the surface of biomaterial and the main functional groups were analysed by EDX and FTIR spectroscopy (NEXUS-650, USA).

### 2.5. Single-factor experiment

#### 2.5.1. Contacting time

In 50 ml Cr(VI) solution (200 mg l^−1^, pH 7), 0.2 g ACS–Fe_3_O_4_ was suspended in a constant temperature shaker (SUKUN, SKY-211B) at 180 rpm, 25°C. Samples were collected at 0, 10, 20, 30, 60, 90, 120, 180, 240, 300, 360 and 420 min.

#### 2.5.2. Cr(VI) concentration

In a series of 50 ml Cr(VI) solutions (20, 50, 100, 200, 400, 600 and1000 mg l^−1^, pH 7) at 25°C, 180 rpm for 24 h, 0.2 g ACS–Fe_3_O_4_ was suspended.

#### 2.5.3. Dosage

Different doses of ACS–Fe_3_O_4_ with concentrations of 2, 4, 6–22, 24 g l^−1^ were added to 50 ml of 200 mg l^−1^ Cr(VI) solution (pH 7) for 24 h in a constant temperature shaker (SUKUN, SKY-211B) at 180 rpm, 25°C.

#### 2.5.4. pH

In 50 ml Cr(VI) solution (200 mg l^−1^) at different pH (ranged from 0–8), which was adjusted by 0.5 mol l^−1^ H_2_SO_4_ or 1 mol l^−1^ NaOH, for 24 h at 180 rpm, 25°C, 0.2 g ACS–Fe_3_O_4_ was suspended.

#### 2.5.5. Stirring rate

Experiments were conducted at 0, 100 and 200 rpm, 25°C with 50 ml Cr(VI) (200 mg l^−1^, pH 7) and 0.2 g ACS–Fe_3_O_4_ for 24 h.

#### 2.5.6. Temperature

Experiments were conducted at 20, 30, 40°C with 50 ml Cr(VI) solution (200 mg l^−1^, pH 7) and 0.2 g ACS–Fe_3_O_4_ for 24 h at 180 rpm.

The filtrate was used to measure the content of Cr(VI) and total Cr.

### 2.6. Taguchi experiment design

The Taguchi method had been widely used as a systematic approach to optimize the design parameters, which can minimize the overall testing time and the experimental costs [24,25]. Using the specially designed orthogonal array, the optimum experiment conditions can be determined.

Accordingly, an analysis of the signal-to-noise (S/N) ratio was applied to evaluate the experimental results. This study was designed to acquire the optimized operational conditions for the maximum Cr(VI) removal percentage; therefore, the HB (higher is best) S/N ratio analysis was adopted.

### 2.7. Optimization study

Six controllable factors were considered, with three levels each. By using JMP 10 (SAS, USA), a Taguchi method array was created (table 2). A series of Cr(VI) solutions (50 ml) were treated with shaking flask test. The treatment time: 120, 180, 240 min; the dosage of adsorbent: 8, 10, 12 g l^−1^; the temperature: 20, 30, 40°C; the pH: 3, 7, 11; the Cr(VI) concentrations: 200, 400, 600 mg l^−1^; the rpm: 0, 100, 200.

**Table 2.** The result of the Taguchi method. The value in bold is the maximum S/N ratio.

	factors						
tests	A	B	C	D	E	F	S/N
1	A1	B1	C1	D1	E1	F1	23.80
2	A1	B1	C1	D1	E2	F2	35.56
3	A1	B1	C1	D1	E3	F3	**36.07**
4	A1	B2	C2	D2	E1	F1	14.64
5	A1	B2	C2	D2	E2	F2	27.13
6	A1	B2	C2	D2	E3	F3	27.52
7	A1	B3	C3	D3	E1	F1	20.37
8	A1	B3	C3	D3	E2	F2	23.47
9	A1	B3	C3	D3	E3	F3	26.13
10	A2	B1	C2	D3	E1	F2	25.91
11	A2	B1	C2	D3	E2	F3	32.85
12	A2	B1	C2	D3	E3	F1	32.85
13	A2	B2	C3	D1	E1	F2	15.76
14	A2	B2	C3	D1	E2	F3	25.22
15	A2	B2	C3	D1	E3	F1	24.31
16	A2	B3	C1	D2	E1	F2	26.95
17	A2	B3	C1	D2	E2	F3	29.90
18	A2	B3	C1	D2	E3	F1	29.49
19	A3	B1	C3	D2	E1	F3	27.79
20	A3	B1	C3	D2	E2	F1	28.19
21	A3	B1	C3	D2	E3	F2	29.07
22	A3	B2	C1	D3	E1	F3	33.71
23	A3	B2	C1	D3	E2	F1	32.42
24	A3	B2	C1	D3	E3	F2	33.77
25	A3	B3	C2	D1	E1	F3	24.36
26	A3	B3	C2	D1	E2	F1	25.07
27	A3	B3	C2	D1	E3	F2	23.87

The analysis of mean statistical approach was conducted to evaluate the optimal conditions.

The details and equations for Taguchi experiment design and optimization study are shown in electronic supplementary material, table S1.

### 2.8. Mechanism study

#### 2.8.1. Sorption isotherms study

A series of Cr(VI) solutions with different initial concentrations were prepared (50, 100, 150, 200 and 300 mg l^−1^). The experiment conditions were: the dose 5 g l^−1^, 24 h, 180 rpm. After equilibrium, *q* (mg g^−1^), weight of Cr removed per unit of dry adsorbent weight, was calculated by

$$q=\frac{C_{0}-C_{e}}{W}V,$$

where *C*_0_ and *C*_e_ (mg g^−1^) are the initial and equilibrium concentrations of Cr(VI), respectively. *V* (l) is the volume of the solution, *W* (g) is the mass of dry adsorbent [26]. Langmuir and Freundlich isotherm models were used to analyse the sorption equilibrium data [27].

#### 2.8.2. Adsorption kinetics study

To investigate the mechanism and characteristics of the adsorption of Cr(VI), pseudo-first-order and pseudo-second-order models were used to test the data [28].

#### 2.8.3. Adsorption thermodynamic study

Thermodynamic parameters, including the free energy change ($\Delta G^{0}$, kJ mol^−1^), enthalpy change ($\Delta H^{0}$, kJ mol^−1^) and entropy change ($\Delta S^{0}$, kJ mol^−1^), were also calculated [28].

The equations for the mechanism study are shown in electronic supplementary material, table S2.

### 2.9. The effect of storage time on Cr(VI) removal

ACS–Fe_3_O_4_ and pure Fe_3_O_4_ were tested for the effect of storage time on Cr(VI) removal. They were stored in vacuum desiccator for 63 days. And the ability of them for Cr(VI) removal from liquid was tested every 7 days under the optimal condition from optimization study.

## 3. Results and discussion

### 3.1. Influence of modification

Both raw and modified ACS showed the ability to remove pollutants from wastewater. The Cr(VI) removal capacity for the native material (ACS) was 1.93 mg g^−1^ dry biomass, and 16.84 mg g^−1^ for ACS–Fe_3_O_4_ (8.72-fold increase compared to ACS). As shown in figure 1, the ability of pure Fe_3_O_4_ and ACS–Fe_3_O_4_ for Cr(VI) removal was much better than that of unmodified materials, and pure Fe_3_O_4_ presented a higher but not significant (*p* < 0.05) removal capacity than ACS–Fe_3_O_4_, indicating ACS–Fe_3_O_4_ had similar adsorption capacity to pure Fe_3_O_4_ at current experiment condition, but better stability as proved later in the paper.

### 3.2. Characterization of biosorbent

#### 3.2.1. Scanning electron microscopy results

**Figure 2.**
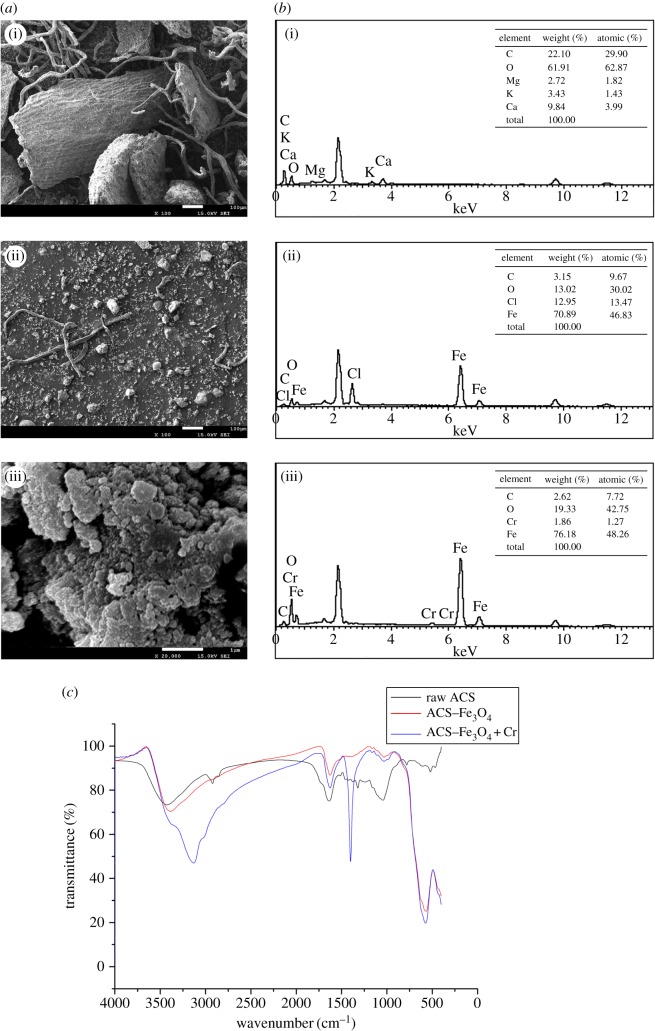
Characterization of the material. (*a*) SEM images of ACS (i), ACS–Fe_3_O_4_ (ii) and nanostructure of ACS–Fe_3_O_4_ (iii). (*b*) EDX spectra of ACS (i), ACS–Fe_3_O_4_ (ii) and ACS–Fe_3_O_4 _+ Cr (iii). (*c*) FTIR spectra of raw *A. cylindracea* substrate material (raw ACS), Fe_3_O_4_-modified *A. cylindracea* substrate material (ACS–Fe_3_O_4_) and after adsorption of Cr on ACS–Fe_3_O_4_ (ACS–Fe_3_O_4 _+ Cr).

As shown in figure 2*a*, before modification, the material was bulky, and its surface was uneven and irregular. However, after modification, the particle was further broken, particle size shrank, and the surface became smoother because of the coating. The modification also provided ACS with nanostructure which was helpful for adsorption. The irregularities of ACS enhanced its ability to adsorb metal ions and also decreased the mass transfer resistance. After modification, the decrease of particle size increased its specific surface area which enhanced the biosorption efficiency.

#### 3.2.2. Energy-dispersive X-ray analysis

The surface of ACS was composed of carbon (22.10 wt%) and oxygen (61.91 wt%) as well as a small amount of Mg, Ca and K before modification (figure 2*b*). After coating, Fe (76.18 wt%) became its main component, verifying the availability of modification. After the treatment, Cr was identified on the surface of ACS–Fe_3_O_4_, confirming the Cr adherence onto absorbent.

#### 3.2.3. Fourier transform infrared analysis

The infrared spectra of ACS, ACS–Fe_3_O_4_ and ACS–Fe_3_O_4_–Cr are demonstrated in figure 2*c*. The broad band observed between 3000 and 3700 cm^−1^ indicated the existence of –OH and –NH groups on both unloaded and Cr-loaded biomaterial. It has been reported [29] that biosorbents normally have intense absorption bands around 3200–3500 cm^−1^. The spectra of Cr-loaded material also displayed absorption peaks at 3126 and 2925 cm^−1^, corresponding to the stretching of C–H bonds of methyne and methylene groups [29,30]. The region between 1690 and 1500 cm^−1^ represented the C≡C stretching in aromatic rings [31]. The peaks observed at 1635 and 1501 cm^−1^ could be attributed to this vibration. The peak observed at 1033 cm^−1^ could be related to the vibration of C–OH in alcohol group and carboxyl [32]. The peak observed at 1395 cm^−1^ represented the vibration of –CH–(CH_3_) [33]. The band at 522 cm^−1^ represents C–N–C scissoring that was only found in protein structures [29], indicating the possible existence of *A. cylindracea* mycelium. However, it was not observed in the spectra of ACS–Fe_3_O_4_ and ACS–Fe_3_O_4_–Cr, indicating the structure of material was changed after modification. The difference of bands between ACS and ACS–Fe_3_O_4_ as well as ACS–Fe_3_O_4_–Cr between 470 and 570 cm^−1^ indicated dramatic change happed to ACS structure after modification.

The FTIR results suggested the binding sites, including –NH_2_, –OH and –COOH on ACS–Fe_3_O_4_, participated in the process.

### 3.3. Results of single-factor experiments

**Figure 3.**
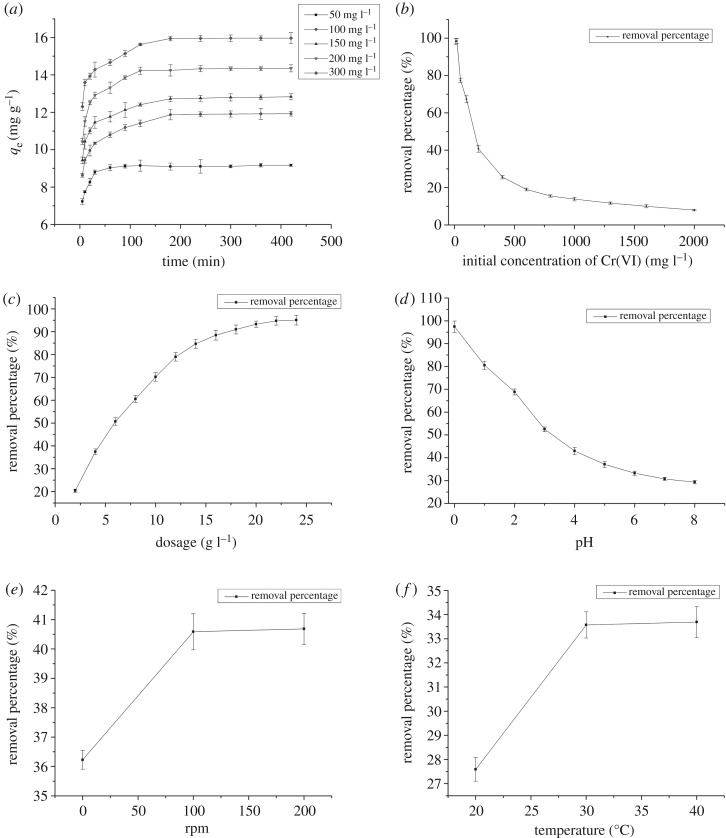
The result of single-factor experiment. Error bars represent the standard deviation of three samples. (*a*) Effect of contacting time. (*b*) Effect of initial concentration. (*c*) Effect of dosage. (*d*) Effect of pH. (*e*) Effect of rpm. (*f*) Effect of temperature.

The results of single-factor experiments are shown in figure 3.

Figure 3*a* shows that when 0.2 g ACS–Fe_3_O_4_ was suspended in a 50 ml Cr(VI) solution (200 mg l^−1^, pH 7) at 180 rpm, 25°C, the adsorption capacity of ACS–Fe_3_O_4_ at different initial concentrations increased fast at the beginning, then slowed down until reaching equilibrium. The adsorption process mainly happening at the start could be attributed to the instantaneous utilization of the most readily available active sites on the material surface. The saturation of the available binding sites slowed down the adsorption speed later. The increase of Cr(VI) concentration led to an increase in the biosorption uptake, so was the equilibrium time. The competition of amount of ions during the adsorption on biosorbent contributed to this increase. Unlike other experiments [28], in the current study, chemical reaction ($\text{C}{\text{r}}^{6+}+3\text{F}{\text{e}}^{2+}\to \text{C}{\text{r}}^{3+}+3\text{F}{\text{e}}^{3+}$) happened along with adsorption process which extended the equilibrium time. After the saturation of the available binding sites, chemical reaction of Cr(VI) and Fe(II) made the main contribution to Cr(VI) removal. The turning points, 120, 180 and 240 min, were chosen as factor levels in optimal experiment.

Profile for Cr(VI) removal when 0.2 g ACS–Fe_3_O_4_ was suspended in a series of 50 ml Cr(VI) solutions (pH 7) at 25°C, 180 rpm for 24 h is shown in figure 3*b*. With the increase of Cr(VI) concentration from 20 to 1000 mg l^−1^, biosorption capacity of ACS–Fe_3_O_4_ increased from 2.46 to 40.08 mg g^−1^ and the removal percentage of Cr(VI) decreased from 98.28% to 7.94%. The interaction between Cr(VI) and biosorbent was improved because of the increase of Cr(VI) concentration, resulting in the improvement of adsorption capacity. However, limited active sites and Fe_3_O_4_ amount on biosorbent surface restricted the biosorption capacity of biosorbent. And at higher ion concentration, the active sites and Fe_3_O_4_ were saturated or exhausted which ended the process. The turning points 200, 400 and 600 mg l^−1^ were chosen as factor levels in optimal experiment.

Figure 3*c* presents the effect of adsorbent dose with ACS–Fe_3_O_4_ suspended in the 50 ml 200 mg l^−1^ Cr(VI) solution (pH 7) for 24 h at 180 rpm, 25°C. With the increase of dosage (2–24 g l^−1^), the removal percentage increased gradually with a slowing trend (from 20.37 to 95.07%). Similar to other reports [32,34], the increase of Cr(VI) removal was due to the increase of active sites and reaction substrate in biosorbent. Further increase of dose concentration did not contribute to the removal, indicating the excessive addition of biosorbent is uneconomical, which was why 8, 10, 12 g l^−1^ were chosen as factor levels in optimal experiment.

The effects of pH are shown in figure 3*d* with 0.2 g ACS–Fe_3_O_4_ suspended in the 50 ml Cr(VI) solution (200 mg l^−1^) for 24 h at 180 rpm, 25°C. With the increase of pH (from 0 to 8), the removal percentage decreased (from 97.43 to 29.33). It has been confirmed that Cr(VI) removal decreased with pH increase [35,36]. The maximal adsorption efficiency happened when the pH was pretty low because solution pH changed the form of the chromium ion, protonation level and the surface charge of the adsorbent [37]. With neutral or alkaline pH, the main form of chromium ion was CrO_4_^2−^. However, Cr(VI) gradually became the main form with the decrease of pH (CrO_4_^2− ^+ 8H^+ ^→ Cr^6+ ^+ 4H_2_O), which increased the competition ability of Cr(VI) with dichromate ion or its hydroxide form, enhancing adsorption efficiency. However, extreme acid condition was not practical, hence acid (pH at 3), neutral (pH at 7) and alkaline (pH at 11) were chosen as factor levels in optimal experiment.

It can be seen in figure 3*e* that removal percentage of Cr(VI) was enhanced (from 36.22 to 40.59) when rpm increased from 0 to 100. However, no significant difference was observed with further increase of rpm. The strengthening of vibration contributed to the contact of biosorbent and ions. And when this vibration reached a certain level, higher stirring rate contributed less to the adsorption. For better investigation of the effect of stirring rate on the adsorption process, three factor levels of 0, 100 and 200 rpm were introduced in optimal experiment.

The temperature presented similar effect as rpm (figure 3*f*). Cr(VI) removal percentage was enhanced from 27.59 to 33.57 when temperature increased from 20 to 30°C, while no significant change happened when temperature increased from 30 to 40°C. The increase of temperature accelerated the molecular movement and contributed to the interaction of adsorbents and solution. However, after reaching a certain level, this acceleration weakened. For better investigation of temperature influence, three factor levels of 20, 30, 40°C were introduced in optimal experiment.

### 3.4. Optimal study

#### 3.4.1. Optimum conditions

According to the Taguchi method, Tests 1–27 were accomplished. The Cr(VI) removal efficiency and the S/N ratio at each test condition were determined. The value in bold in table 2 represents the maximum value of S/N ratio. In electronic supplementary material, table S3, the maximum value of ${\left(M\right)}_{\mathrm{factor}=I}^{\mathrm{level}=i}$ among all six factors in three levels was bolded, which indicated the optimization condition for Cr(VI) removal. The optimum conditions were: 40°C, pH 3, Cr(VI) concentration 200 mg l^−1^, adsorbent 12 g l^−1^, rpm 200 and 240 min.

**Figure 4.**
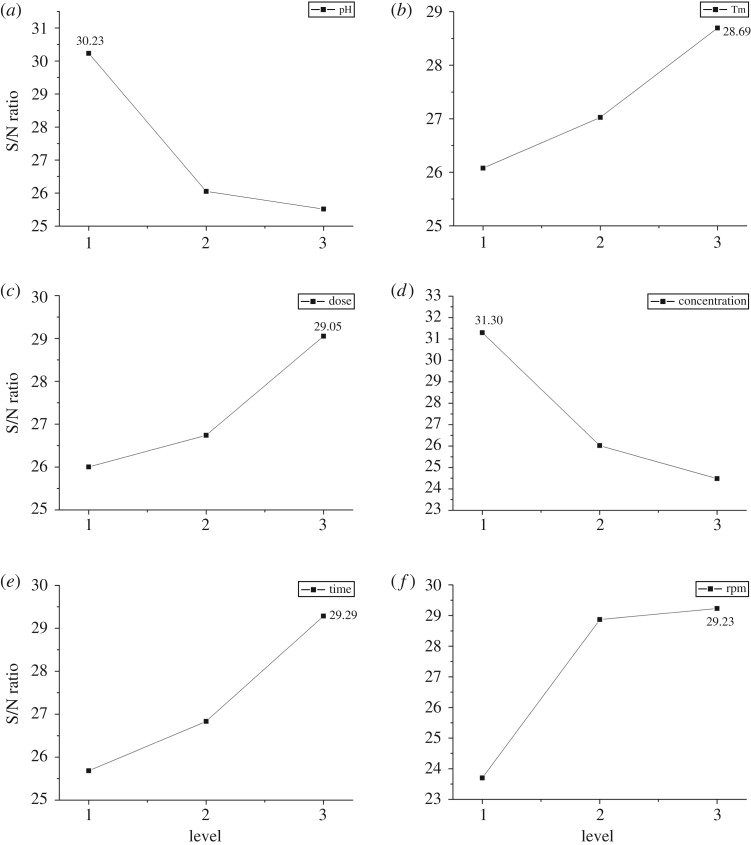
The effects of all controllable factors on S/N ratio.

Figure 4*a* shows the effect of solution pH on S/N ratio. With the lowest pH at level 1 (pH 3), S/N was a peak at 30.23. And the lowest S/N of 25.51 happened at level 3.

With the enhancement of temperature, S/N ratio value increased (figure 4*b*), indicating that higher ambient temperature influenced the adsorption more.

It is well defined in figure 4*c* that the S/N ratio reached the peak at 29.05, when the dosage was 12 g l^−1^. While the lowest S/N ratio emerged when the dosage was 8 g l^−1^. This was because the increase of adsorbent dosage led to the increase of adsorption sites, improving pollutant removal efficiency.

When it came to Cr(VI) concentration (figure 4*d*), the highest S/N ratio value (31.30) occurred at 200 mg l^−1^ and decreased with its increase, indicating that the increase of metal concentration played weaker role in adsorption mainly because of the limited adsorption sites on biosorbent.

According to figure 4*e*, the highest S/N ratio value was 29.29 when treatment time was 240 min, and the lowest value was 25.68 with treatment time at 120 min. This result indicated that although the adsorption process mainly happened in the early phase as shown in figure 3*a*, it continued with time increasing. And the final equilibrium state prolonged with the increase of Cr(VI) concentration. At given condition in optimal study with Cr(VI) concentration between 200 and 600 mg l^−1^, the equilibrium could be around 240 min where the highest S/N ratio value was reached. Therefore, the optimum treatment time should be 240 min with Cr(VI) concentration between 200 and 600 mg l^−1^.

As shown in figure 4*f*, the highest S/N ratio value was 29.23 at 200 rpm, which presented no significant difference from 100 rpm, indicating that beyond a certain level, the increase in rpm had a weak influence on Cr(VI) removal.

#### 3.4.2. Verification test

In real industry process, the parameters of the polluted liquid could be complex, and the concentration of Cr in wastewater could be different [38]. To verify the availability of optimal conditions and feasibility of ACS–Fe_3_O_4_ in practice, a verification test was conducted using tannery wastewater. The detailed experiment process is presented in the electronic supplementary material and the result is shown in electronic supplementary material, table S4. After treatment, 98.35% of Cr(VI) and 95.60% of total Cr were removed. The treatment also contributed to the decrease of COD, NH_4_–N and Cl, verifying the feasibility of the adsorbent and optimized adsorption conditions from this study in practical use.

### 3.5. Contribution of each factor

The results of SS_F_ and ${\bar{R}}_{k}^{F}$ are shown in table 3 and electronic supplementary material, table S5, respectively. SS_T_, the total sum of squares, was 19110.170. The variance of error, *V*_E_ (99.521), was also obtained. In the end, the contribution ratio of each factor was determined and shown in table 3. Initial concentration of Cr(VI) was the most influential factor in the process, whose contribution ratio was 33.310%. Solution pH had second highest significant influence with contribution ratio at 13.010%. The stirring rate, dosage and treatment time posed minor influence on the process, and the contribution ratios were 9.952%, 5.879% and 3.861%, respectively. The temperature posed adverse but small (−0.384%) effect on treatment. However, in practical application, metal concentrations and solution pH could not be efficiently controlled without high cost in practical application, hence more attention should be paid to controllable factors like rpm or dose concentration.

**Table 3.** Contribution ratio of each factor.

factor	DOF_F_	SS_F_	ρ%	SS_T_	*V*_E_
A: *T*_m_	3	125.583	−0.384	19110.170	99.521
B: pH	3	2685.368	13.010		
C: concentration	3	6564.610	33.310		
D: dose	3	1322.563	5.879		
E: rpm	3	2100.938	9.952		
F: time	3	936.978	3.861		

### 3.6. Mechanism study

#### 3.6.1. Biosorption isotherms

Adsorption isotherms provide important information that reveals the equilibrium relationship between the adsorbate concentration in the liquid phase and the solid phase at a constant temperature. Langmuir and Freundlich models, which correspond to homogeneous and heterogeneous adsorbent surfaces, respectively [34], were chosen to describe the equilibrium characteristics of this study. The average regression coefficients (*r*^2^) of the Langmuir model (0.779–0.977) were higher than those of the Freundlich model (0.716–0.922) (table 4A), indicating the Langmuir model was more suitable to describe the sorption process, and monolayer adsorption occurred on a heterogeneous adsorbent surface. Moreover, the value of *b* was 0.0048 (0<b<1), which confirmed the favourable uptake of Cr(VI).

**Table 4.** The results of mechanism study.

(A) isotherm model constants for Cr(VI) adsorption					
isotherm model		293 K	303 K		313 K
Langmuir	*Q*_max_ (mg g^−1^)	34.602	42.553		27.855
	*b* (l mg^−1^)	0.0048	0.0037		0.0053
	RL	0.410–0.807	0.474–0.844		0.388–0.792
	*r*^2^	0.779	0.910		0.977
Freundlich	*K*_F_ (mg g^−1^(mg l^−1^)^1/*n*^)	3.194	3.803		3.279
	*n*	4.248	5.189		3.751
	*r*^2^	0.716	0.764		0.922

(B) kinetic parameters for Cr(VI) adsorption					
	Cr(VI) concentration (mg l^−1^)				
kinetic model	50	100	150	200	300
pseudo-first order					
*Q*_e, exp_ (mg g^−1^)	9.450	12.165	13.246	17.211	15.236
*Q*_e, cal_ (mg g^−1^)	1.816	3.243	3.704	6.256	3.506
*k*_1_ (min^−1^)	0.0076	0.0124	0.0099	0.0060	0.014
*r*^2^	0.805	0.952	0.910	0.957	0.952
pseudo-second order					
*Q*_e, cal_ (mg g^−1^)	9.479	12.240	13.333	17.33102	15.314
*k*_2_ (g mg^−1^ min^−1^)	0.0077	0.0059	0.0042	0.001759	0.0069
*r*^2^	0.9992	0.9996	0.9989	0.9962	0.9999

(C) thermodynamic parameters of Cr biosorption on ACS–Fe_3_O_4_					
			ΔG (kJ mol^−1^)		
biosorbent	ΔH (kJ mol^−1^)	ΔS (kJ mol^−1^)	283.15 K	293.15 K	303.15 K
ACS–Fe_3_O_4_	4.805	0.030	−3.816	−3.942	−4.421

#### 3.6.2. Kinetics of Cr(VI) biosorption

Two models (pseudo-first-order and pseudo-second-order) were used to test the data (table 4B). With a higher *r*^2^ value, the pseudo-second-order model was better fitted to the data than the pseudo-first-order model. Moreover, its calculated adsorption capacity closely fitted the experimental data. Therefore, the present adsorption system followed a predominantly pseudo-second-order kinetics model. A similar result for the treatment of waste water has also been reported in other works [39,40].

#### 3.6.3. Thermodynamics of biosorption

As shown in table 4C, the values of the free energy change $\left(\Delta G^{0}\right)$ were negative, indicating the feasibility and spontaneous nature of the adsorption process. The positive value of $\Delta S^{0}$ indicated the increased randomness at the solid/solution interface during the adsorption, suggesting the good affinity of Cr(VI) towards the adsorbent and significant changes occurred in the internal structure of the biosorbent during biosorption. Furthermore, the positive $\Delta H^{0}$ value confirmed that the adsorption was an endothermic process.

### 3.7. The effect of storage time on Cr(VI) removal

**Figure 5.**
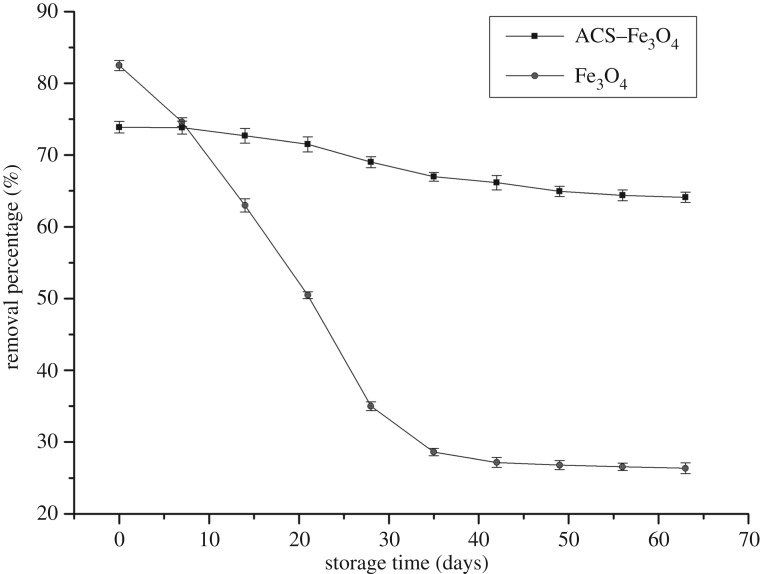
The effect of storage time on Cr(VI) removal. Error bars represent the standard deviation of three samples.

The ability of ACS–Fe_3_O_4_ and pure Fe_3_O_4_ for Cr(VI) removal from liquid was tested every 7 days during 63 days' storage, with the optimal conditions (40°C, pH 3, Cr(VI) concentration at 200 mg l^−1^, dosage at 12 g l^−1^, rpm at 200, and treatment time was 240 min) derived from optimization study (figure 5). The removal percentage of pure Fe_3_O_4_ decreased rapidly during 63 days from 82.49 to 26.36% with a quick and then slowing trend. However, the removal ability of ACS–Fe_3_O_4_ decreased slightly compared with pure Fe_3_O_4_, which proved the protection effect of biomaterial supporting on Fe_3_O_4_. And ACS–Fe_3_O_4_, as a magnetic biomaterial, showed strong stability in ambient environment.

## 4. Conclusion

With uneven surface and polyporous structure, ACS–Fe_3_O_4_ was proved to be an efficient biosorbent for Cr(VI) removal. FTIR analysis revealed that functional groups, including –NH_2_, –OH and –COOH, provided binding sites for Cr(VI). Nanostructure and chemical property of Fe_3_O_4_ also contributed to Cr(VI) removal. Under the optimum conditions, ACS–Fe_3_O_4_ can remove 73.88% of Cr(VI). Removal percentages of Cr(VI) and total Cr were 98.35 and 95.6, respectively, when applying in real tannery wastewater. Among all the controllable factors, initial Cr(VI) concentration and solution pH posed largest contribution to adsorption, indicating pollutant removal could be enhanced by adjusting pollutant concentration and liquid acidity. The Langmuir model was more suitable to describe the sorption process in this study, indicating monolayer adsorption occurred on the heterogeneous adsorbent surface. This research not only proposed a novel, economic and eco-friendly bio-adsorbent with environmental stability for metals ion removal but also put forward an ideal option for biomaterial application and waste management.

## Supplementary Material

The equations for Taguchi experiment design and mechanism study as well as detailed analysis data

## Data accessibility

Data are available from the Dryad Digital Repository: http://dx.doi.org/10.5061/dryad.k3j3d [41].
