# Supplementary material for: Fe3O4 nanoparticle-coated mushroom source biomaterial for Cr(VI) polluted liquid treatment and mechanism research
Source: R Soc Open Sci. 2018 May 9;5(5):171776. doi: 10.1098/rsos.171776 (PMC5990751; doi:10.1098/rsos.171776)
Supplement: The equations for Taguchi experiment design and mechanism study as well as detailed analysis data [file rsos171776supp1.docx]

**Supplementary Material** **for**

Fe_3_O_4_ nanoparticle-coated mushroom source biomaterial for Cr(VI) polluted liquid treatment and mechanism research

Can Wang, Huakang Liu, Zizhao Liu, Yufeng Gao, Bin Wu, Heng Xu*^^[[1]](#footnote-1)^^

*Key Laboratory of Bio-resources and Eco-environment (Ministry of Education), College of Life Science, Sichuan University, Chengdu, Sichuan 610064, China*

Details for verification test

The tannery wastewater was obtained from a tannery from Zhengxing, Tianfu New Area, Chengdu, Sichuan, China (30°25′28″ N, 104°0′47″ E). Before experiment, the wastewater was filtrated to remove suspended solids. And parameters of the wastewater including COD_Cr_, NH_4_-N, Cl and Cr(VI) were determined according to Chinese National Standard GB/T 11914, HJ/T 195, HJ/T 84, GB/T 7467, separately. Cr_total_ was determined by FAAS.

The test was conducted at optimal treatment condition derived from Taguchi methods except for initial Cr(VI) concentration (240min, 40°C, pH 3, dosage at 12 g L^-1^, rpm at 200) with 50mL wastewater in a 100ml conical flask. The pH was adjusted to 3 with 0.5mol L^-1^ H_2_SO_4_. The parameters of wastewater were shown in STable 4. According to Chinese National Standard GB 30486-2013 Discharge standard of water pollutants for leather and fur making industry, after treatment by ACS-Fe_3_O_4_, all the parameters were below the standard values except COD_Cr_, indicating ACS-Fe_3_O_4_ was efficient for the practical treatment of tannery wastewater.

# Table Legends ( STable for Supplementary Material Table):

STable 1 The equations for Taguchi experiment design and optimization study.

STable 2 Models equations of biosorption isotherms, kinetics and thermodynamic studies.

STable 3. S/N ratio for each factor in each level.

STable 4 Parameters of tannery wastewater before and after treatment.

STable 5 The average of removal efficiency results of a certain factor in the $k$th level ($\overline{R}_{k}^{A}$,$\overline{R}_{k}^{B}$, $\overline{R}_{k}^{C}$,$\overline{R}_{k}^{D}$) and the average of total removal efficiency ($\overline{R}_{T}$).

STable 1 The equations for Taguchi experiment design and optimization study

| Factor | Equation | Symbols representation |
| --- | --- | --- |
| $\frac{S}{N}$, the signal-to-noise (S/N) ratio | $\frac{S}{N}=-10log\left( \frac{1}{n}\sum_{i=1}^{n} \frac{1}{R_{i}^{2}} \right)$ | where n is the number of experiments under the same conditions, R represents the Cr(VI) removal efficiency |
| R, the Cr(VI) removal efficiency (removal percentage) | $R=\left( \frac{C_{0}-C_{e}}{C_{0}} \right)\times100$ | where C_0_ and C_e_ separately represent the initial and equilibrium concentrations of Cr(VI) (mg L^-1^). |
| $\left( M \right)_{Factor =I}^{Level =i}$, the mean of the S/N ratio of each controllable factor at a certain level | $\left( M \right)_{Factor =I}^{Level =i}=\frac{1}{n_{Ii}}{\sum_{j = 1}^{n_{Ii}} \left[ \left( \frac{S}{N} \right)_{Factor =I}^{Level =i} \right]}_{j}$ | $n_{Ii}$represents the number of appearances of factor I in the level I under the influence of factors, $\left( \frac{S}{N} \right)_{Factor =I}^{Level =i}$ represents the S/N ratio of factor I in level i, j represents the appearance sequence of prior variable in Table 2. |
| $\rho$, the contribution rate of each factor | $\rho=\frac{{SS}_{F} -\left( {DOF}_{F}V_{E} \right)}{{SS}_{T}}\times100$ | where ${DOF}_{F}$ represents the degree of freedom for each factor, which was obtained by subtracting one from the number of the level of each factor (L). |
| ${SS}_{T}$, the total sum of squares, | ${SS}_{T}=\sum_{j=1}^{m} \left( \sum_{i=1}^{n} {R_{i}}^{2} \right)_{j}-mn\left( \bar{R}_{T} \right)^{2}$ | where $\bar{R}_{T}=\sum_{j=1}^{m} \left( \sum_{i=1}^{n} R_{i} \right)_{j}\div\left( mn \right)$, m represents the number of experiments carried out in this study, and n represents the number of repetitions. |
| ${SS}_{F}$, the factorial sum of squares | ${SS}_{F}=\frac{mn}{L}\sum_{k=1}^{L} \left( \overline{R}_{k}^{F}-\overline{R}_{T} \right)^{2}$ | where $\overline{R}_{k}^{F}$ is the average value of the removal efficiency of a certain factor in the $k$th level. |
| $V_{E}$, the variance of error | $V_{E}=\frac{{SS}_{T}-\sum_{F=A}^{D} {SS}_{F}}{m\left( n-1 \right)}$ | - |

STable 2 Models equations of biosorption isotherms, kinetics and thermodynamic studies

| Model | Equation | Symbols representation |
| --- | --- | --- |
| Pseudo-first order | $\log\left( q_{e}-q_{t} \right)=log q_{e}-\frac{k_{1}}{2.303}t$ | Where $q_{e}$ and $q_{t}$ (mg g^-1^), are the amounts of Cr(VI) removed at equilibrium and at any time t (min) per unit weight of adsorbent, respectively; while $k_{1}$ (min^-1^) and $k_{2}$ (g mg^-1^ min^-1^), which are calculated from the slopes of $\log(q_{e}-q_{t})$*versus t* and $\frac{t}{q_{t}}$ versus *t*, are the rate constants of pseudo-first order and pseudo-second order adsorption, respectively. |
| Pseudo-second order | $\frac{t}{q_{t}}=\frac{t}{k_{2}{q_{e}}^{2}}+\frac{t}{q_{e}}$ |  |
| Langmuir isotherms model | $\frac{C_{e}}{q}=\left( \frac{1}{q_{m}b} \right)+\left( \frac{1}{q_{m}} \right)C_{e}$ | Where $q$ (mg g^-1^) is the weight of Cr(VI) removed per unit of dry adsorbent weight at equilibrium, $C_{e}$ (mg L^-1^) is the equilibrium Cr(VI) concentration. $q_{m}$ (mg g^-1^）is the maximum monolayer capacity, $b$ (L mg^-1^) is the adsorption affinity onto the adsorption sites and it is related to energy of adsorption, both of them are Langmuir constants, which can be calculated through the slope and intercept of the linear plot with $\frac{C_{e}}{q}$ versus $C_{e}$. |
| Freundlich isotherm model | $\ln q=\ln k_{f}+\left( \frac{1}{n} \right)\ln C_{e}$ | Where $q$ (mg g^-1^) is the weight of Cr(VI) removed per unit of dry adsorbent weight at equilibrium, $C_{e}$ (mg L^-1^) is the equilibrium Cr(VI) concentration. $k_{f}$ (mg g^-1^) represents the sorption capacity and $n$ (l/mg) represents the sorption intensity. $k_{f}$, the Freundlich constant can be calculated through the slope and intercept of the linear plot with $\ln q$ versus $\ln C_{e}$. |
|  | $K_{0}=\frac{C_{e}(adsorbent)}{C_{e}(solution)}$ | Where $R$ is the universal gas constant (8.314 J K^-1^ mol^-1^), T the absolute temperature ($K$) and $K_{0}$ the distribution coefficient. |
| Thermodynamic study |  |  |
|  | $\Delta G^{0}=-RT\ln K_{0}$ |  |
|  |  |  |
|  | $\Delta G^{0}=\Delta H-T\Delta S$ |  |

| STable 3. S/N ratio for each factor in each level | | | | | | | | | | |
| --- | --- | --- | --- | --- | --- | --- | --- | --- | --- | --- |
| Factor/level | [${(S/N)}_{Factor}^{Level}$] _j_ | | | | | | | | | ${(M)}_{Factor}^{Level}$ |
|  | j=1 | j=2 | j=3 | j=4 | j=5 | j=6 | j=7 | j=8 | j=9 |  |
| A1 | 23.80 | 35.56 | 36.07 | 14.64 | 27.13 | 27.52 | 20.37 | 23.47 | 26.13 | 26.08 |
| A2 | 25.91 | 32.85 | 32.85 | 15.76 | 25.22 | 24.31 | 26.95 | 29.90 | 29.49 | 27.03 |
| A3 | 27.79 | 28.19 | 29.07 | 33.71 | 32.42 | 33.77 | 24.36 | 25.07 | 23.87 | **28.69** |
| B1 | 23.80 | 35.56 | 36.07 | 25.91 | 32.85 | 32.85 | 27.79 | 28.19 | 29.07 | **30.23** |
| B2 | 14.64 | 27.13 | 27.52 | 15.76 | 25.22 | 24.31 | 33.71 | 32.42 | 33.77 | 26.05 |
| B3 | 20.37 | 23.47 | 26.13 | 26.95 | 29.90 | 29.49 | 24.36 | 25.07 | 23.87 | 25.51 |
| C1 | 23.80 | 35.56 | 36.07 | 26.95 | 29.90 | 29.49 | 33.71 | 32.42 | 33.77 | **31.30** |
| C2 | 14.64 | 27.13 | 27.52 | 25.91 | 32.85 | 32.85 | 24.36 | 25.07 | 23.87 | 26.02 |
| C3 | 20.37 | 23.47 | 26.13 | 15.76 | 25.22 | 24.31 | 27.79 | 28.19 | 29.07 | 24.48 |
| D1 | 23.80 | 35.56 | 36.07 | 15.76 | 25.22 | 24.31 | 24.36 | 25.07 | 23.87 | 26.00 |
| D2 | 14.64 | 27.13 | 27.52 | 26.95 | 29.90 | 29.49 | 27.79 | 28.19 | 29.07 | 26.74 |
| D3 | 20.37 | 23.47 | 26.13 | 25.91 | 32.85 | 32.85 | 33.71 | 32.42 | 33.77 | **29.05** |
| E1 | 23.80 | 14.64 | 20.37 | 25.91 | 15.76 | 26.95 | 27.79 | 33.71 | 24.36 | 23.70 |
| E2 | 35.56 | 27.13 | 23.47 | 32.85 | 25.22 | 29.90 | 28.19 | 32.42 | 25.07 | 28.87 |
| E3 | 36.07 | 27.52 | 26.13 | 32.85 | 24.31 | 29.49 | 29.07 | 33.77 | 23.87 | **29.23** |
| F1 | 23.80 | 14.64 | 20.37 | 32.85 | 24.31 | 29.49 | 28.19 | 32.42 | 25.07 | 25.68 |
| F2 | 35.56 | 27.13 | 23.47 | 25.91 | 15.76 | 26.95 | 29.07 | 33.77 | 23.87 | 26.83 |
| F3 | 36.07 | 27.52 | 26.13 | 32.85 | 25.22 | 29.90 | 27.79 | 33.71 | 24.36 | **29.29** |

STable 4 Parameters of tannery wastewater before and after treatment

(Units: mg L^-1^ except pH)

| Parameters | Limit value | Before | After | Removal percentage |
| --- | --- | --- | --- | --- |
| COD_Cr_  NH_4_-N | 300  70 | 2641.82  124.15 | 892.41  59.67 | 63.75%  51.94% |
| Cl | 4000 | 5157.34 | 3096.20 | 39.97% |
| Cr(VI) | 0.20 | 5.46 | 0.09 | 98.35% |
| Cr_total_  pH | 1.50  6-9 | 19.78  7.03 | 0.87  4.36 | 95.60% |

STable 5 The average of removal efficiency results of a certain factor in the $k$th level ($\overline{R}_{k}^{A}$,$\overline{R}_{k}^{B}$, $\overline{R}_{k}^{C}$,$\overline{R}_{k}^{D}$) and the average of total removal efficiency ($\overline{R}_{T}$)

| Level | $\overline{R}_{k}^{A}$ | $\overline{R}_{k}^{B}$ | $\overline{R}_{k}^{C}$ | $\overline{R}_{k}^{D}$ | $\overline{R}_{k}^{E}$ | $\overline{R}_{k}^{F}$ | $\overline{R}_{T}$ |
| --- | --- | --- | --- | --- | --- | --- | --- |
| 1 | 26.995 | 37.502 | 41.025 | 31.125 | 21.166 | 24.129 | 28.441 |
| 2 | 27.933 | 27.080 | 28.903 | 24.404 | 31.024 | 28.317 |  |
| 3 | 29.977 | 24.653 | 19.307 | 23.221 | 32.716 | 32.460 |  |

1. *Corresponding author. Tel: +86 28 85414644; Fax: +86 28 85418262

   E-mail address: [xuheng64@sina.com](mailto:xuheng64@sina.com)(H. Xu).

   The first two authors contributed equally to this work and should be considered co-first authors. [↑](#footnote-ref-1)
